# The need to update Ethiopia’s national health and medicine policies: essential tools for informed decision-making in healthcare and the pharmaceutical sectors

**DOI:** 10.3389/fpubh.2025.1533710

**Published:** 2025-04-25

**Authors:** Ewunetie Mekashaw Bayked

**Affiliations:** Department of Pharmacy, College of Medicine and Health Sciences (CMHS), Wollo University, Dessie, Ethiopia

**Keywords:** National Health Policy, National Drug Policy, National Medicine Policy, update, monitoring, evaluation

## Abstract

**Background:**

Health policy involves decisions at national, state, and local levels to achieve healthcare goals, with medicine policy being a critical component that requires integration and potential reform. The World Health Organization (WHO) recommends periodic monitoring and evaluation of the National Medicine Policy (NMP) under the National Health Policy (NHP), ideally every 2 to 3 years or comprehensively every 4 to 5 years.

**Objectives:**

This perspective aims to urge the Ministry of Health (MOH) of Ethiopia to update its national health and medicine policies to address emerging health issues, advancements in medical treatment, and global health agendas. It also aims to initiate a name change for Ethiopia’s “NDP” to “NMP” to focus on medicines for therapeutic purposes while avoiding confusion with the term “drug.”

**Methods:**

A descriptive critical evidence synthesis was used to identify indicators and situations for monitoring, evaluating, and updating national health and medicine policies. The author presented published evidence to support the perspective that Ethiopia’s national health and medicine policies need to be updated.

**Results:**

Ethiopia’s national health and medicine policies have remained unchanged for over three decades, despite their critical role in guiding healthcare decision-making and reflecting political commitment to advancing healthcare goals through regular monitoring and evaluation. This underscores an urgent need to update these policies and periodically monitor and evaluate them at prescribed intervals: every 2 to 3 years for minor changes or every 4 to 5 years comprehensively. Additionally, the title “National Drug Policy (NDP)” should be changed to “NMP” to better reflect its focus on safe, effective, and approved medicines for healthcare, while avoiding negative associations with the general term “drugs”.

**Conclusion:**

Given the dynamic nature of the health and pharmaceutical sectors, it is crucial for Ethiopia to urgently update the NHP and NMP and change the NDP title to “NMP” to eliminate ambiguity, emphasize approved medicines, and align with global best practices.

## 1 Introduction

### 1.1 Background

Ethiopia’s health system is divided into three tiers: primary (including health posts, health centers, and district hospitals), secondary (general hospitals), and tertiary (regional and national hospitals) (1). This nation, one of the oldest states in the Horn of Africa, is grappling with poor health outcomes due to decades of lack of revised national health and medicine policies, weak healthcare infrastructure, and low government spending (2).

The WHO defines health policy as decisions, plans, and actions taken by institutions and organizations at national, state, and local levels to achieve specific healthcare goals (3). An NMP is an essential part of health policy; it cannot be developed in isolation. It should align with broader health objectives (4), be integrated into the national health system (5), and undergo periodic monitoring and evaluation (6).

Policy monitoring is a continuous process that involves stakeholder engagement, progress evaluation, legislative endorsement, and outcome evaluation to ensure policy effectiveness (7). Evaluation involves appraising needs, midterm effectiveness, and reviewing achievements for future lessons using monitoring indicators (5, 8).

The WHO has developed indicators to monitor an NMP in all countries using low-cost, non-complex methods (5, 9). These indicators are divided into four categories: 31 background information indicators (quantitative data), 50 structural indicators (qualitative data), 38 process indicators (quantitative data), and 10 outcome indicators. These indicators can be modified or removed to suit specific national contexts, ensuring effective monitoring and evaluation of NMPs (9, 10).

### 1.2 Rationale

Support from interest groups, concerned stakeholders, favorable macroeconomic conditions, technical expertise, and committed individuals within the MOH can help overcome barriers and advance an effective NMP (5). This underscores that the responsibility for amending the NHP and NMP extends beyond the government to academic institutions, professional associations like the Ethiopian Pharmaceutical Association (EPA), international organizations like the WHO, Non-Governmental Organizations (NGOs), pharmaceutical policy experts, pharmaceutical industries, the private sector, and the public (11).

The WHO Constitution’s eighth principle reinforces this, emphasizing that “informed recommendation and active cooperation from the public are of utmost importance in improving people’s health.” This highlights that both those who directly implement changes and those who challenge existing values with evidence-based arguments are crucial drivers of policy change (12).

In light of this scenario, as a researcher and expert in social and administrative pharmacy (SAPh), I offer my evidence-based recommendation on the importance of monitoring and evaluating health and medicine policies in Ethiopia. To do so, I conducted an extensive review of the MOH of Ethiopia^1^ and the Ethiopian Food and Drug Administration Authority (EFDA, http://www.efda.gov.et/) websites to determine if the national health and medicine policies have been revised or if there have been movements toward updating them.

Utilizing a descriptive critical evidence synthesis approach, as mentioned above, I analyzed key indicators and situations relevant to monitoring, evaluating, and updating the policies. To support my argument, I also incorporated recently published evidence, highlighting emerging health issues, technological advancements in medical treatments, and shifts in global health priorities. This evidence forms the basis of my perspective, emphasizing the urgent need for Ethiopia to align its health and medicine policies with current and future health challenges.

### 1.3 Objectives

This perspective aims to urge the MOH of Ethiopia to update its national health and medicine policies to address emerging health issues, advancements in medical treatment, and global health agendas. It also aims to initiate a name change for Ethiopia’s “NDP” to “NMP” to focus on medicines for health promotion, disease prevention, treatment, cure, and rehabilitation, while avoiding confusion with the broad term “drug,” which can include non-medical substances.

## 2 Perspectives

The healthcare and pharmaceutical sectors are constantly changing due to globalization (13, 14) and many other factors. Among the key areas driving change are technological and scientific advancements, including the rise of Artificial Intelligence (AI) (15–17), digital health and/or health technologies (18, 19), gene therapy (20–22), the discovery of new treatments such as biosimilars (23, 24), and nutraceuticals (25).

Global health initiatives and policies are also important drivers of change in the sectors. Initiatives like One Health (26), Healthy People 2030 (27), and Universal Health Coverage (UHC) (28) or health economics (pharmacoeconomics) policies (29–32) are set to be important global health priorities. Programs such as the Sustainable Development Goals (SDGs) (33, 34), along with international frameworks like Trade-Related Aspects of Intellectual Property Rights (TRIPS) and conferences like the International Conference on Drug Regulatory Authorities (ICDRA) and the International Conference on Harmonization (ICH), are guiding the global health agenda (35). Regional initiatives, such as the African Medicines Agency (AMA) (36) and the Health Extension Program (HEP) of Ethiopia (37), also have significant influence.

In addition, the sectors are increasingly expected to address global and emerging health threats. These include emerging and re-emerging diseases (38), Neglected Tropical Diseases (NTDs) (39, 40), and the growing concern of Antimicrobial Resistance (AMR) (41, 42).

Another important aspect is the ongoing shift in healthcare systems and practices. This includes the need to integrate traditional medicine into the modern health system (43), which helps create more inclusive, culturally sensitive healthcare approaches (44). Mental health issues (45), the treatment of drug use disorders (46), and the need for specialized care for the aging population (47) are also critical areas of focus.

Furthermore, ethical and social considerations are central to healthcare discussions. Issues such as euthanasia (physician-assisted suicide—PAS) (48, 49) and *In Vitro* Fertilization (IVF) (50) continue to prompt debates about medical ethics and the rights of patients.

The above emerging issues and WHO indicators thus underscore the need for countries, which did not revise, to update their health and medicine policies. However, while the NMP should be periodically monitored, evaluated, or amended every 2 to 3 years (5, 6, 51), comprehensively every 4 to 5 years (52), Ethiopia’s NMP, developed in November 1993 (53), has not been revised, failing to address the ever-changing nature of the pharmaceutical sector. Similarly, the NHP, developed in September 1993 (54), has not been updated to reflect global and local changes in the health sector reforms.

As a result, the Ethiopian NHP and NMP are failing to align with current local and global health objectives. The Ethiopian Public Health Institute (EPHI) 2021–22 Final Report, published in July 2023, states that the NHP is under revision to address sociodemographic, epidemiologic, and economic shifts in Ethiopia. This revision aligns with Ethiopia’s goal of becoming a middle-income country, as well as commitments to UHC and the SDGs in the health sector. As to the report, the revised policy has undergone consultations and is awaiting ratification by the Council of Ministers (55). Yet, the report does not mention updates to the NMP, and no public documents on updated NHP are currently available.

The delay in updating the NHP and NMP may be due to factors like conflict and political instability for the current government of Ethiopia, particularly since 2018, driven by political entrepreneurs (56). The 2019 WHO review of 40 years of primary healthcare implementation also revealed that conflict and political instability have significantly hindered PHC efforts (57), leading to policy agenda abandonment, suboptimal development, and implementation failures (58). In fact, health policy processes often face obstacles like fragmentation, weak advocacy, unclear agendas, insufficient evidence, lack of consultation, and corruption (58). Like the NHP, developing NMP is also challenging due to political will, resource constraints, opposition, and corruption (5).

Nevertheless, as rationalized above, support from interest groups, shared stakeholder values, favorable macroeconomic conditions, technical expertise, and committed MOH individuals can overcome these challenges (5). Despite the barriers mentioned, monitoring and evaluation using such facilitators are thus crucial for ensuring policies meet goals, address public health needs, adapt to challenges, improve accountability, resource allocation, and health outcomes. Revising the NHP and NMP, therefore, is essential for guiding regulatory decisions in healthcare and the pharmaceutical sectors, respectively (27). Hence, Ethiopia needs to update its NHP along with the NMP to adapt to global and local health challenges and improve health outcomes.

Moreover, it is recommended that the title “NDP” be changed to “NMP” to reduce ambiguity and better reflect the policy’s focus on promoting safe, effective, and high-quality essential medicines. Because, while “drug” broadly refers to any chemical substance, including harmful ones, “medicine” specifically denotes substances developed and approved to treat, cure, or prevent diseases, emphasizing their therapeutic purpose. This distinction clarifies the policy’s intent, aligns it with healthcare objectives, and ensures clarity for stakeholders. Additionally, using the term “medicine” aligns with international best practices, strengthening the policy’s commitment to public health and global consistency (59).

## 3 Conclusion

Health policy encompasses decisions, plans, and actions at national, state, and local levels to achieve healthcare goals, with medicine policy being a key component. Periodic monitoring and evaluation of the NMP under the NHP are essential to keep align with the evolving health and pharmaceutical sectors. However, Ethiopia’s national health and medicine policies have remained unchanged for over three decades, despite recommendations to ideally update them every 2 to 3 years or comprehensively every 4 to 5 years. Therefore, it is urgent to update these policies and periodically monitor and evaluate them according to the prescribed intervals. Additionally, changing the title from “NDP” to “NMP” is crucial to eliminate ambiguity, better reflect the focus on safe, effective, and approved medicines, and align with global best practices, while avoiding negative associations with the general term “drugs.”

## Data Availability

The information relevant to the perspective is included in the article, further inquiries can be directed to the corresponding author.

## Footnotes

^1^ https://www.moh.gov.et/
